# Dental Management of a Tunisian Child with Sanjad-Sakati Syndrome

**DOI:** 10.1155/2022/9585460

**Published:** 2022-04-22

**Authors:** Farah Chouchene, Aymen Ben Haj Khalifa, Fatma Masmoudi, Ahlem Baaziz, Fethi Maatouk, Hichem Ghedira

**Affiliations:** ^1^Pediatric and Preventive Dentistry Department, Faculty of Dental Medicine of Monastir, Laboratory of Biological, Clinical and Dento-Facial Approach, University of Monastir, Monastir, Tunisia; ^2^Department of Dental Anatomy, Faculty of Dental Medicine, University of Monastir, Monastir, Tunisia

## Abstract

Sanjad-Sakati syndrome (SSS) is a rare autosomal recessive congenital disorder. The present case report is aimed at describing the orofacial manifestations and dental management of a 4-year seven-month-old, Tunisian boy with SSS. The patient has typical dysmorphic facial features and growth retardation. Intraoral examination revealed micrognathic mandible and maxilla, an arched palate, and small dental arches with an open bite. All the maxillary and mandibular teeth were decayed due to the poor oral hygiene, plaque accumulation, and enamel hypoplasia. Oral rehabilitation involved pulpotomies and root canal therapies on decayed teeth. Resin composite restorations were performed on maxillary and mandibular incisors, and stainless-steel crowns were placed on maxillary and mandibular first and second primary molars. Dental treatment of children with SSS should improve their quality of life and their general health. Undeveloped dental arches associated with dental anomalies as well as learning deficit make very difficult of the oral rehabilitation of such patients.

## 1. Introduction

Sanjad-Sakati syndrome (SSS) is a rare autosomal recessive disorder that may affect both genders. This syndrome known as hypoparathyroidism-retardation-dysmorphism (HRD) syndrome has been described mainly in the Middle East regions with a higher prevalence among Arab population [1–3].

This disorder was described for the first time in 1991 by Sanjad et al. among a group of Saudi children [4].

Children with SSS may exhibit congenital hypoparathyroidism, with severe intrauterine, postnatal growth, mental retardation, intellectual disability, and dysmorphic features [2, 3, 5].

Most of the reported cases of SSS have been associated with parental consanguinity. This rare autosomal syndrome has been also predominantly reported in patients of Arab origin [5]. This may be explained by the norms of consanguineous marriage which are dominant and widespread in these regions, thus resulting in the retention and increased rate of homozygote autosomal recessive genetic disorders in these populations [1].

SSS is characterized by the presence of congenital severe hypocalcemia, hypoparathyroidism, and hyperphosphatemia leading to early onset of epileptic seizures. The neonatal hypocalcemia usually causes apnea, convulsions, and tetany in these patients [2, 5, 6].

Children suffering from SSS have typical dysmorphic craniofacial features including microcephaly, narrow face, large floppy ears, deep-set eyes, thin lips, depressed nasal bridge, micrognathia, and abnormal dentition [1, 3–7].

During infancy and childhood, given their immune deficits, children with SSS generally presented with recurrent infections [4, 5].

The distinct clinical features of this rare disorder are related to mutations in the gene-encoding tubulin specific chaperone E (TBCE; 604934), located on chromosome 1q42-43 which is listed in Online Mendelian Inheritance in Man (OMIM) #241410 [1, 2, 8].

This syndrome presents several oral manifestations which could help to correctly diagnose and distinguish SSS from other syndromes associated with congenital hypoparathyroidism, such as Kenny-Caffey, Barakat, and DiGeorge syndromes [1, 2, 8].

Malocclusions associated to a high-arched palate, hypodontia, microdontia, taurodontism, and dental defects such as hypoplastic enamel may be reported in children with SSS [5, 9].

Learning disabilities, endocrinopathy, and seizure disorders could complicate dental treatment in these young patients [1].

This report is aimed at describing the orodental/facial manifestations of a 4-year seven-month-old male patient diagnosed with SSS and discussing the dental management considerations.

## 2. Case Report

### 2.1. Presentation

A 4-year seven-month-old, Tunisian boy was referred to the Pediatric Dentistry Department at the Faculty of Dental Medicine of Monastir (Tunisia) complaining of pain related to poor oral and dental condition.

### 2.2. Medical History

The patient' medical history revealed that the young boy was initially diagnosed with SSS by his pediatrician who relied on clinical features, consanguineous factor, and biochemical measures to confirm the diagnosis.

The patient was born to consanguineous parents and had no other siblings.

He had history of full-term birth by caesarean section with a birth weight of 2 kg, a birth height of 42 cm, and a head circumference of 31 cm.

The patient's medical history reported also episodes of tetany and convulsions associated with signs and symptoms of hypocalcemia from the 15th day of birth. The seizures lasted a few minutes and were resolved without intravenous antiepileptic drug administration.

The initial patient's biochemical examinations showed hyperphosphatemia (2.5 mmol/L), hypocalcemia (1.8 mmol/L), and very low levels of parathyroid hormone (4.2 pg/L) (Table 1).

Vitamin D and oral calcium preparations were prescribed by the patient's pediatrician during clinical follow-ups, but due to recurrence of hypocalcemia, second episodes of seizures were observed at the age of 13 months.

A16 months, the pediatrician general examination revealed characteristic facial dysmorphism with microcephaly (−3 standard deviation [SD]) and severe growth retardation (−4 SD for height, −3 SD for weight).

At this age, the patient height was 80 cm and weight was 14 kg.

The general examination demonstrated also intrauterine growth retardation (IUGR), oligohydramnios, and fetal distress. His hands and feet were small with thin and short digits, which gave him a very short stature.

No congenital heart abnormality was reported.

The genetic testing revealed 12 bp (155–166 del) within the TBCE gene in exon 3 confirming the diagnosis of SSS.

The neurological examination revealed an intellectual disability, with a mental retardation, a psychomotor delay, and a severe speech delay which made the dental examination and treatment very difficult to perform.

### 2.3. Clinical Examination

**Figure 1:**
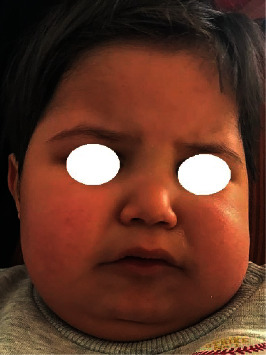
Head and neck examination.

Extraoral head and neck examination confirmed the diagnosis of SSS and reported typical craniofacial features of this syndrome such as microcephaly, large set ears, deep-set eyes, prominent forehead, beaked nose, thin lips, and marked philtrum (Figure 1).

Intraoral examination revealed micrognathic mandible and maxilla, an arched palate, and small dental arches with an open bite.

**Figure 2:**
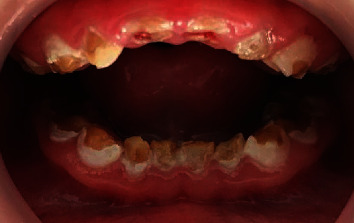
Intraoral view (preoperative features).

All the maxillary and mandibular teeth were severely decayed due to the poor oral hygiene, the plaque accumulation, and the enamel hypoplasia (Figure 2).

### 2.4. Dental Management

Radiographic examinations were not possible due to the patient behavior.

The proposed treatment plan was discussed and approved by the patient's parents.

Written informed consent was obtained from the child's parents for all imaging exams, treatment modalities, and data publications.

The full dental treatment was planned and summarized in Table 2.

A pulpotomy was performed on teeth #62 and #52 after removal of the coronal pulp using a sterile excavator. The pulp chamber was then rinsed with normal saline, and the bleeding was controlled using a small cotton pellet.

A zinc oxide-eugenol mixed to a thick consistency was then applied and condensed over the imputed pulp of the prepared cavity.

Finally, the rest of the cavity was partially filled with type IX glass ionomer cement (GIC) (Riva self-cure, SDI Australia) and then restored with resin composite (3M™ FZ100 MP-USA).

A root canal therapy was performed on teeth #51, #61, #54, #55, #64, #65, #74, #75, #84, and #85, by the same pediatric dentist using the same procedure.

After administration of local anesthesia, coronal access was performed with a #02 round high-speed carbide burr without isolation using a rubber dam since isolation was difficult to perform for this patient.

Then, after identification of the canal orifices, the root canals were manually prepared using stainless steel manual K-files (Dentsply-Maillefer, Ballaigues, Switzerland) in a step back manner through size #35 by quarter-turn-pull technique. The root length was determined approximately given the impossibility of taking X-rays. The pulp chamber was abundantly irrigated with 2.5% sodium hypochlorite.

All the root canals were dried using paper points and sealed using zinc oxide-eugenol. Finally the access cavities were restored with type IX GIC (glass ionomer cement) (Riva self-cure, SDI Australia).

Composite resin restorations (3M™ FZ100 MP-USA) were placed on the maxillary and mandibular incisors and stainless-steel crowns (3M™ ESPE™-USA) on all maxillary and mandibular primary molars.

All the dental treatments were carried out over several sessions under local anesthesia because the patient's parents refused categorically general anesthesia.

**Figure 3:**
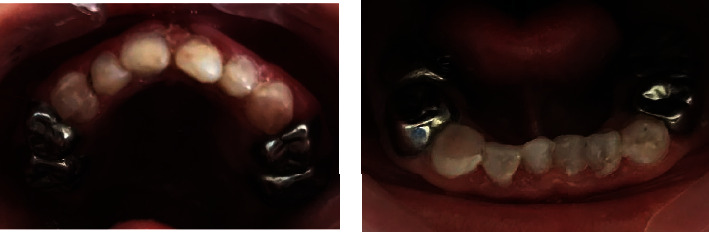
Postoperative restorative treatments.

Figure 3 shows the postoperative restorative treatments.

Dietary advice with instructions on oral hygiene was given for the parents, and regular follow-up visits were scheduled every 6 months and showed a very satisfactory clinical condition of all the restorative treatments.

Regular follow-up visits were scheduled, and dental prophylaxis was performed at each visit.

## 3. Discussion

This disorder was first reported in children living in Saudi Arabia and then has been reported in other countries due to globalization and migration [1].

The number of published clinical cases of patients with SSS has increased, and more than 90 cases have been reported in the literature.

Almost all the patients were from Arab countries (Morocco, Egypt, Oman, Jordon, Kuwait, Sudan, Qatar, Saudi Arabia, and Tunisia), and only three were from non-Arab origin (Belgium and India) [2, 6, 7, 9–11].

For the best of our knowledge, this was the first reported case describing dental management of a Tunisian child with SSS.

The deep rooted norm of consanguineous marriage in Arab population has been widely blamed as a predisposing factor for autosomal recessive diseases such as SSS, and this was confirmed in the current clinical report [9, 12].

SSS molecular pathology has been shown to be due to mutations in the TBCE gene on chromosome 1q42-q43. All the affected patients of Arab origin have shown homozygosity for a 12 bp (155-166 del) deletion in exon 3 of the TBCE gene [8, 11].

The fact that this mutation has so far been reported only in SSS patients from Arab families suggests a long-standing founder effect [11].

In Tunisia, all the reported cases [7], including our patient, demonstrated the existence of the same mutation.

It is distinctly possible that the origin of these mutations is the Middle East, and that during the propagation of Islam in North Africa by the invaders of Banu Hilal in the 7th century, these mutations were introduced in Tunisia [3, 11].

SSS patients exhibit special dysmorphic features as well as severe growth retardation both intrauterine and postnatal such as the following disorders: familial hypoparathyroidism, Kenny-Caffey syndrome, and DiGeorge syndrome. But the latter is distinguished by the absence of cardiac and skeletal abnormalities, ophthalmic pathology, or alteration of immune functions that can manifest themselves in the other diseases. To differentiate these syndromes, genetic tests are necessary [2–4, 6, 10].

In the present reported case, different characteristic features of SSS, such as short stature, small hands and feet, learning disability, and facial features including prominent forehead, microcephaly, deep-set eyes, marked philtrum, large floppy ears, beaked small nose, thin upper lip, retrognathism, and atypical teeth with dental caries, have been reported in the young patient.

Oral manifestations of this syndrome were poorly described and were not well reported in the literature. Recognition of these oral manifestations facilitates the diagnosis of SSS and helps distinguish it from other syndromes [1, 5, 9, 13].

The severe dental caries reported in the young patient could be due to simultaneous presence of several factors but it is mainly attribute to enamel hypoplasia. In fact, enamel defects increased the retention of dental plaque, thus increasing the risk of dental caries.

Untreated hypocalcemia and hypoparathyroidism diagnosed at a very young age could be the cause of such enamel defects [1, 5].

The patient cooperation, oral hygiene, salivary function, and composition could also been affected by this syndrome which may be considered as additional factors increasing the risk of developing carious lesions.

In addition to these factors, frequent infections, sugary drugs, and cariogenic dietary habits increased the caries risk of this patient.

The combination of these different factors may contribute to the alteration of patient general health and oral hygiene [1, 5].

In the present report, the patient had undergone dental treatment under local anesthesia since the parents refused categorically general anesthesia.

Al-Malik [5], Wasersprung et al. [13], and El Batawi [9] reported dental management of SSS cases that went well under general anesthesia despite the fact that the airway management under general anesthesia in such patients may be complicated by the dwarfism, the retrognathic mandible, the seizures, and the endocrine abnormalities [14].

Considering the behavioral problems associated with this syndrome, the dental management was very difficult.

The treatment plan is aimed essentially at preserving and restoring all the decayed teeth there by rehabilitating both the aesthetics and the functions.

Due to the limited cooperation of the patient as well as his very limited mouth opening, all the restorative procedures were performed without radiographic examination.

A clinical check-up after 6 months showed a very satisfactory clinical condition of all the steel crowns.

No long-term follow-up of patients with SSS dental management has been found in the literature [9].

In the case of SSS reported by Wasersprung et al. [13], the absence of 12 permanent teeth on the radiological examination was reported. In our clinical case, the absence of germs from the permanent teeth was difficult to confirm since no radiological examination could be performed.

The possibility of reencountering anodontia may be encountered in SSS, and for this, reason restoration of primary teeth using performed crowns instead of dental filling is recommended, and this the same therapeutic approach was adopted by El Batawi [9].

It is very important that pediatricians provide parents of children with SSS with good education on nutritional and oral hygiene habits taking into consideration the child masticatory efficiency as well as intellectual abilities.

Parents should also be informed about the possible repercussions of their child's general conditions on the oral health.

Regular follow-up visits should be scheduled, and dental prophylaxis should be performed at each visit.

## 4. Conclusion

Dental treatment of children with SSS should improve the child's quality of life as well as his general health. It is also essential to improve masticatory function. Therefore, it is recommended to use stainless steel crowns.

Undeveloped dental arches associated with dental anomalies as well as learning deficit make oral rehabilitation of these patients very difficult.

Treatments must be long-lasting, and multidisciplinary approaches are necessary for successful dental treatment.

## Figures and Tables

**Table 1:** The initial laboratory investigations of the patient.

Reference range	Calcium (2.2–2.6 mmol/L)	Phosphate (0.8–1.45 mmol/L)	Parathyroid hormone (15–65 pg/L)
The patient	1.8 mmol/L	2.5 mmol/L	4.2 pg/L

**Table 2:** Summary of the provided treatments.

Tooth number	Treatment provided
52, 62	Pulpotomy and resin composite restoration
51, 61	Root canal treatment and resin composite restoration
54, 55, 64, 65, 74, 75, 84, and 85	Root canal treatment and stainless-steel crown
53, 63, 73, 72, 71, 81, 82, and 83	Resin composite restorations

## Data Availability

All data generated and analysed which are related this work are included in this published article.
